# Hyperhomocysteinemia and Pulmonary Embolism in a Young Male

**DOI:** 10.7759/cureus.7818

**Published:** 2020-04-24

**Authors:** Olga Kovalenko, Ahmad N Kassem, Melissa Jenkins

**Affiliations:** 1 Obstetrics and Gynecology, University Hospitals Cleveland Medical Center, Case Western Reserve University School of Medicine, Cleveland, USA; 2 Internal Medicine, MetroHealth Medical Center, Case Western Reserve University, Cleveland, USA; 3 Infectious Disease, MetroHealth Medical Center, Cleveland, USA

**Keywords:** thrombosis, pulmonary embolism, small bowel resection, anticoagulation, hyperhomocysteinemia, homocysteine, folate, vitamin b12, cteph, saddle embolus

## Abstract

The association of hyperhomocysteinemia with thrombosis has provoked debate in the medical literature. Although studies have found associations between moderate homocysteine elevations and thrombotic events, others dispute this relationship. We present herein the case of a 24-year-old male who presented with unprovoked bilateral submassive pulmonary emboli. Extensive hypercoagulability workup was notable for an elevated homocysteine level, in addition to low vitamin B12 and folate levels. Of note, the patient had a history of small bowel resection after trauma, which may have contributed to the aforementioned metabolic derangements, potentially increasing his risk for thrombosis and interfering with the efficacy of his anticoagulation.

## Introduction

The association of hyperhomocysteinemia with thrombosis has triggered debate in the medical literature. While meta-analyses and prospective studies have found associations between moderate homocysteine elevations with recurrent thromboembolic events, other studies have disputed this relationship [1-7]. We present herein an interesting case that would add to the discussion regarding the association between the risk for thrombosis and hyperhomocysteinemia.

## Case presentation

A 24-year-old male presented to the emergency department with three weeks of worsening bilateral pleuritic chest pain with no known inciting factors. He had developed progressively worsening dyspnea on exertion, and had an isolated episode of hemoptysis. The patient admitted to daily tobacco and alcohol use, in addition to occasional marijuana use. Of note, the patient’s mother had a pulmonary embolism (PE) in her late 30s. Two years prior to presentation, he had a prolonged hospitalization after a motor vehicle accident requiring multiple surgical interventions, one of them resulting in the resection of 60 cm of his jejunum. He had no other known past medical history and took no medications. On physical exam, the patient was in apparent discomfort; he had a blood pressure of 133/77 mmHg, a heart rate of 98 beats per minute, a respiratory rate of 22 breaths per minute and a pulse oximetry read 92% oxygen saturation on room air. The remainder of his exam, including lung examination, was unremarkable, except for an abdominal surgical scar.

**Figure 1:**
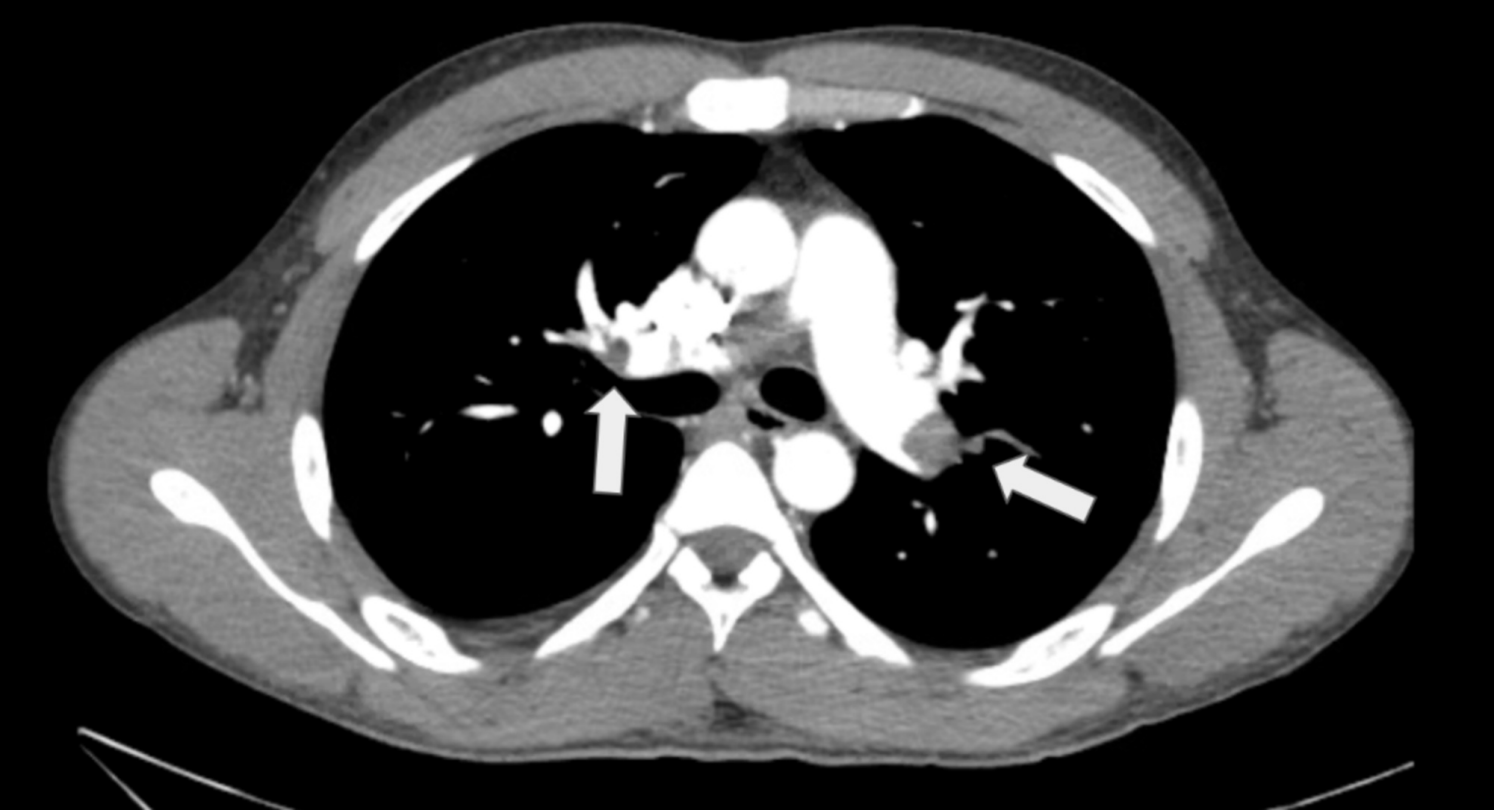
Pulmonary angiography with evidence of filling defects bilaterally in pulmonary vasculature, as noted with arrows.

**Figure 2:**
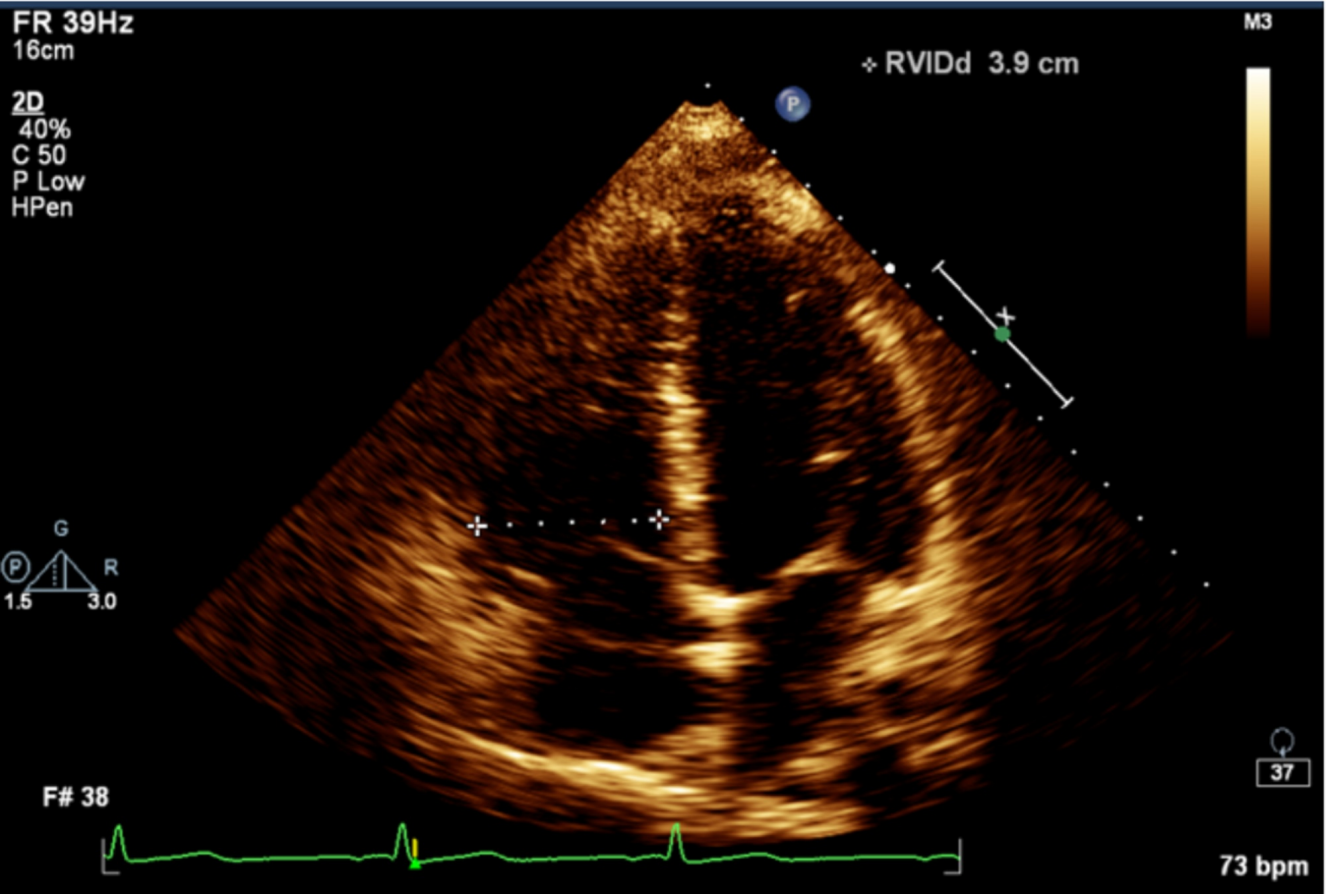
Right ventricular strain as demonstrated by enlarged right ventricle (shown by dashed line) on a four-chamber view of echocardiogram.

Initial laboratory workup included a normal troponin, blood urea nitrogen, creatinine and serum electrolytes. Complete blood count showed a mean corpuscular volume of 104 without anemia and no other abnormalities. EKG showed sinus tachycardia, while chest x-ray showed no abnormalities. A Wells score of 4 was calculated, suggesting moderate risk of PE, after which a D-dimer of 1,371 ng/mL was obtained. B-type natriuretic peptide level (BNP) was 288 pg/mL (normal range). Pulmonary angiography showed extensive bilateral PE (Figure 1), and transthoracic echocardiogram showed right ventricular strain consistent with submassive PE (Figure 2).

Given the patient’s significant clot burden and the unprovoked nature of his PE, extensive workup for thrombophilia was performed. Factor V Leiden, prothrombin mutation, cardiolipin antibody, lupus anticoagulant, anti-B2 glycoprotein, protein C and protein S were all within normal limits. Homocysteine level was 41.3 μmol/L (normal 4.0-13.7 μmol/L). Workup for macrocytosis was notable for low vitamin B12 (72 pg/mL) and folate (2.1 ng/mL) levels. He was treated with systemic unfractionated heparin infusion and was subsequently switched to rivaroxaban. He was discharged in stable condition, with vitamin B12 and folic acid supplementation, in addition to anticoagulation with rivaroxaban. He reported feeling better during an office visit two weeks later. 

The patient presented to another institution three months after the first PE we described above with a saddle PE. He was diagnosed with chronic thromboembolic pulmonary hypertension (CTEPH) for which he underwent a successful bilateral pulmonary endarterectomy; unfortunately, the procedure was complicated by a pericardial effusion requiring pericardiocentesis. His anticoagulation was changed to warfarin after the second thrombotic event. The exact cause of the patient’s thrombophilia remains unclear. 

## Discussion

**Figure 3:**
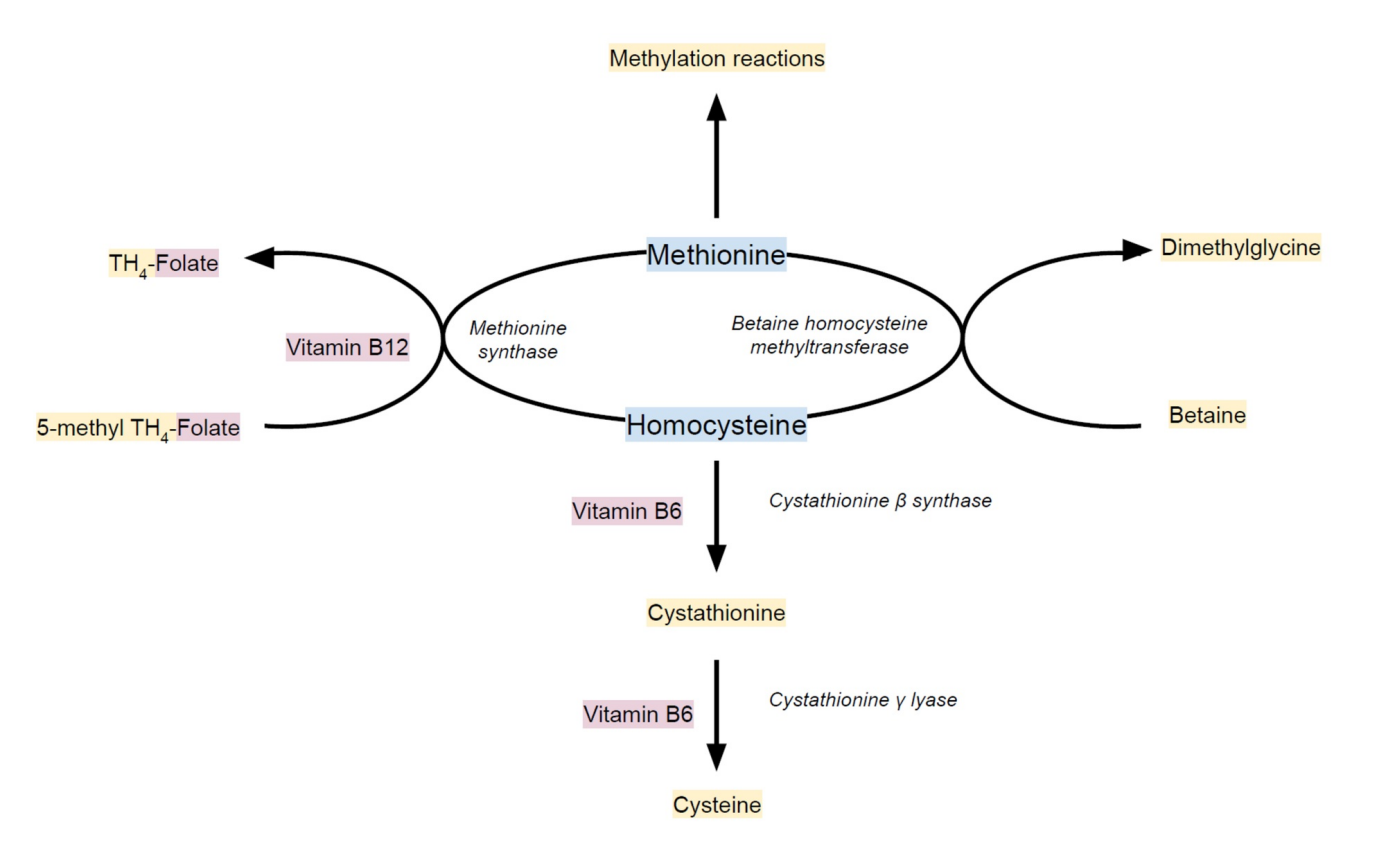
The conversion of homocysteine to the essential amino acid methionine. The conversion occurs through two pathways: (1) methionine synthase catalyzes the methylation of homocysteine by capturing a methyl group from the folate-dependent one-carbon pool (5-methyltetrahydrofolate), requiring vitamin B12 and (2) betaine homocysteine methyltransferase catalyzes the donation of a methyl group to homocysteine from betaine (N,N,N-trimethylglycine). Catabolism of homocysteine to cysteine through transsulfuration occurs via two vitamin B6-dependent enzymes. TH_4_: tetrahydro

Homocysteine, an intermediary amino acid formed from the conversion of methionine to cysteine, is metabolized through transsulfuration and remethylation reactions (Figure 3), requiring vitamins B6 and B12, respectively [4]. Risk factors for hyperhomocysteinemia include methylenetetrahydrofolate reductase (MTHFR) mutations, vitamin B6, vitamin B12 or folate deficiencies, chronic kidney disease, certain medications and tobacco smoking [8]. Previous case reports have described cases of PE, with or without deep vein thrombosis, associated with hyperhomocysteinemia [9,10]. One prospective cohort study found that elevated homocysteine levels are associated with unprovoked, first venous thromboembolic events in middle-aged and older women [5]. Another meta-analysis found hyperhomocysteinemia to be a risk factor for venous thromboembolic events, especially in patients below the age of 60 years [1]. However, another study challenges the relationship between hyperhomocysteinemia and thromboembolism, citing confounding lifestyle-related factors, such as smoking status, BMI and physical activity [3]. 

The uniqueness of the case we presented highlights risk factors for the development of hyperhomocysteinemia and thrombosis in our patient. Folate and vitamin B12 deficiency are known to cause elevations in homocysteine levels [11]. One hypothesis in the case of our patient is whether the small bowel resection he underwent two years prior to his PE, in combination with excessive alcohol use, contributed to poor absorption of B vitamins [12]. Given the family history significant for PE in the patient's mother, genetic testing was performed. Testing showed that he was heterozygous for the C677T variant and normal for the A1298C variant of the MTHFR gene, which usually does not increase the risk of thrombosis. Although not previously reported, it may be possible that even minimal enzyme inactivity may be significant when combined with his other risk factors for the development of hyperhomocysteinemia. Brattström et al. have reported that vitamin B12 replacement reduced homocysteine levels when elevated in the setting of vitamin B12 deficiency [13]. Our patient’s serum homocysteine level was not re-checked after vitamin B12 replacement. Whether B vitamin replacement to reduce homocysteine levels truly decreases the risk of thrombosis is questionable. Two clinical trials demonstrated that despite successfully lowering homocysteine levels through vitamins B6 and B12 supplementation, the risk for symptomatic or recurrent venous thromboembolism is not decreased [6,7].

Despite the use of rivaroxaban, our patient developed a saddle embolus and CTEPH after several months of treatment. This raises questions about the choice of anticoagulation in patients with altered gastrointestinal (GI) anatomy. The absorption of rivaroxaban, the drug of choice in this case initially, occurs in the upper GI tract, and has been reported to be appropriate after Roux-En-Y surgery [14]. However, in cases of poor response to direct oral anticoagulants in patients with disrupted GI anatomy, and after ensuring proper compliance and use, it is reasonable to consider a vitamin K antagonist, since therapeutic levels can be monitored via international normalized ratio measurements [15]. Parenteral anticoagulation can also be utilized, if needed.

## Conclusions

Ultimately, this case illustrates the interplay of genetic, iatrogenic, environmental and social factors leading to metabolic derangements and thrombosis, as well as the wide differential in unprovoked PE. It also provides further evidence in favor of the association between hyperhomocysteinemia and thrombosis. 
